# In Silico Molecular Docking Studies of Phytocompounds From Coleus Amboinicus Against Glucokinase

**DOI:** 10.7759/cureus.34507

**Published:** 2023-02-01

**Authors:** Soosai Marian Anthony Ammal, Sai Sudha, Durairaj Rajkumar, Adhithya Baskaran, Gunasekaran Krishnamoorthy, Madurai Kannan Anbumozhi

**Affiliations:** 1 Department of Anatomy, Karpaga Vinayaga Institute of Medical Sciences and Research Centre, Maduranthagam, IND; 2 Department of Pathology, Sree Balaji Medical College and Hospital, Chennai, IND; 3 Department of Oral Pathology, Adhiparasakthi Dental College and Hospital, Chennai, IND; 4 Department of Medical Biochemistry, College of Medical and Health Sciences, Dambi Dollo University, Oromia Region, ETH

**Keywords:** torbangun, glucokinase, molecular docking, coleus amboinicus, diabetes mellitus

## Abstract

Diabetes is one of the most prevalent metabolic illnesses that can be fatal, and it is the ninth-largest cause of mortality worldwide. Even though there are effective hypoglycemic medications available for the treatment of diabetes, researchers continue to look for a medication that is more effective and has fewer adverse effects by focusing on various metabolic components such as enzymes, transporters, receptors. The enzyme Glucokinase (GCK), which is present mainly in the liver and beta cells of the pancreas, is involved in maintaining blood glucose homeostasis. Hence, the present in silico study is designed to determine the interaction between GCK and compounds (ligands) of *Coleus amboinicus*. In the current docking investigation, we discovered that important residues, including ASP-205, LYS-169, GLY-181, and ILE-225, significantly influence in ligand binding affinity. Docking tests of these compounds with target proteins revealed that this is a suitable molecule that docks well with the target of diabetes treatment. In conclusion, we believe that the compounds of caryophyllene have anti-diabetic activity based on the present study.

## Introduction

One of the four hexokinase isozymes, Glucokinase (GCK), converts glucose to glucose-6-phosphate in the hepatic and pancreatic cells [1]. The perfect glucose sensor and rate-limiting enzyme GCK regulates the rate at which glucose triggers insulin production [2]. GCK activities regulate the rate of glycogen synthesis and utilization in the liver according to Non-Mechaelis-Menton kinetics, demonstrating that GCKs are not controlled by the reaction product glucose-6-phosphate [3]. GCK is mixed with glucose and the enzyme stimulator glucokinase in its crystal structure, which has a palm-shaped topology with two domains of different sizes, the big and small (GCKA). The enormous gap that separates the two domains acts as the binding site for the phosphorylation of glucose. An accessible hydrophobic allosteric pocket is 20 distances from the catalytic site when the kinase is bound to glucose in its "closed" catalytically active state. In the unbound form of glucose (also known as "open form"), the hydrophobic pocket is concealed between the competing big and small domains. Allosteric sites are where GCKAs connect, and they immediately activate GCKs [4]. Because type 2 diabetes mellitus (T2DM) patients have impaired insulin action and secretion, increasing hepatic glucose production and decreased glucose-induced release from pancreatic cells, which makes it different from other types of diabetes. Despite the efforts of several research organizations, no single oral anti-diabetic drug can provide adequate, long-term glycemic control [5]. Although the combination of different classes of hypoglycemic agents provides good blood sugar control than the single agent, the risk of drug adverse effects increase [6]. Consequently, there is a growing need for novel medications that are both effective and safe. GCK's strong impact on glucose homeostasis and the fact that activating it lowers blood glucose levels regardless of the cause of hyperglycemia suggests that it may be a suitable therapeutic target for T2DM [7]. Numerous research groups have shown that small-molecule glucokinase enhancers could enhance glycemic control via a dual mechanism of improved hepatic glucose metabolism and increased pancreatic insulin production [8, 9]. Consequently, the ability of GCKA to affect multiple cell types, such as muscle cells and adipocytes, may increase its efficacy and make it a useful tool in the management of diabetes.

The use of plant-based treatments to treat metabolic illnesses like diabetes is growing in popularity. Numerous plants with high flavonoid content are a hidden gold mine for treating diabetes. Torbangun is a flowering plant (Coleus amboinicus Lour). Torbangun leaf extract possesses antioxidant effects, according to research [10]. Understanding how glucokinase interacts with the molecules in Coleus amboinicus is the aim of this study. This study will advance knowledge of the glucokinase activation mechanism and pave the road for the creation of novel GCKAs that could specifically stimulate GCK in the treatment of T2DM.

## Materials and methods

Protein preparation

Protein Data Bank (PDB id: 1V4S) provided the 3D structure of glucokinase in the pdb format. Using Autodock tools, solvents, unusual ligands, and residues were removed from protein macromolecules before they have been kept in the pdb format. Hydrogen atoms have been added to macromolecules to improve them, and they were then recorded in the pdbqt format [11].

Ligand molecules

Sixteen bioactive compounds from Coleus amboinicus were obtained from literature searches (Table 1). The ligands were downloaded in the sdf format from PubChem (https://pubchem.ncbi.nlm.nih.gov/). The compounds were then improved using PyRx (version 0.8) by energetically converting the ligands into the most stable configurations (MMFF94) [12].

**Table 1:** List of selected compounds from Coleus amboinicus

Sl. No	Compound Name
1	alpha-Pinene
2	beta-Caryophyllene
3	alpha-Terpineol
4	beta-Phellandrene
5	beta-Pinene
6	beta-Selinene
7	Carvacrol
8	Caryophyllene_oxide
9	Eucalyptol
10	Eugenol
11	gamma-Terpinene
12	Methyleugenol
13	p-Cymene
14	Terpinolene
15	Thymol
16	alpha-Pinene

Molecular docking

The flexible docking procedure proposed by Trott and Olson was used to carry out the molecular docking with a few minor adjustments. As a result, Python Prescription 0.8, a package that incorporates Auto Dock Vina, was used to conduct the docking analysis of the identified chemicals with our chosen proteins. For the proteins, partial charge and atom type (PDBQT) files have been created (with their earlier formed PDB files as inputs). All of the ligand's bonds were allowed to rotate freely, which made the receptor stiff. Except for the grid box, which was altered in accordance with the active sites of protein molecules, many other parameters were retained at their default values. After the molecular docking investigations were finished and ten combinations for each protein-ligand complex for all phytoconstituents were constructed, text files containing the score data were also made for manual comparison. The conformation with the lowest binding energy (BE, kcal/mol) and root mean square deviation was found to be the ideal docking site (RMSD). To obtain more precise and trustworthy results, a docking exhaustiveness of 10 was chosen for this in-silico experiment, and the number of modes was fixed at 10. After that, PyMOL and the Discovery Studio visualizer were used to construct, display, and analyze the interaction between ligands and proteins [13].

## Results

AutoDock Vina in PyRx 0.8 was used to perform the molecular docking study. The energy binding values of the chemical components of Coleus amboinicus were shown in Table 2. Caryophyllene oxide, beta-Caryophyllene, Thymol, and beta-Selinene had the strongest glucokinase affinity.

**Table 2:** Molecular docking results of glucokinase with selected bio compounds

S.No.	Compound Name	Binding energy kcal/mol	Hydrogen bond interaction
1	Caryophyllene_oxide	-6	-
2	beta-Caryophyllene	-5.7	LYS-169 (Pi –alkyl) ILE-225 ( Pi –alkyl
3	Thymol	-5.4	GLY-181(H-Bond) ASP-205 (Pi-Anion) LYS-169 (Pi –alkyl) ILE-225 (Pi –alkyl)
4	beta-Selinene	-5.3	LYS-169 (Pi –alkyl) ILE-225 ( Pi –alkyl

## Discussion

In the pancreas and liver, GCK catalyzes the first and rate-limiting step of glycolysis, which is the conversion of glucose to glucose 6-phosphate [14]. GCK is considered to be the body's primary glucose sensor since changes in its activity modify the threshold for glucose-stimulated insulin generation from pancreatic beta-cells [15,16]. Unlike the other hexokinases, GCK is not sensitive to feedback suppression by physiological amounts of its product glucose 6-phosphate [17-19]. GCK is largely produced in the liver's hepatoparenchymal cells and pancreatic β-cells in humans [20]. GCK controls the rate of insulin secretion inside pancreatic cells to maintain glucose homeostasis, whereas GCK contributes to glycogen synthesis in the liver [21]. Numerous disease symptoms caused by alterations in the human gck locus highlight the need for more precise control over GCK activity in both tissues.

The pharmaceutical industry is very interested in creating GCK activators due to their significance in glucose metabolism and illness [22-25]. Numerous lead compounds that either directly or indirectly stimulate GCK in vivo have been discovered [25]. However, no practical therapeutic agent has yet to be discovered as a result of these attempts. The control of GCK is complex, and various novel regulatory strategies have been identified. Alternate and tissue-specific promoters control GCK transcription and gene expression to various degrees [26-30]. Insulin and glucose, among other hormones and metabolites, control the transcriptional level of GCK [17,31-34].

The different illness symptoms brought on by alterations in the human gck locus emphasize the need for careful regulation of GCK activity in both organs. Permanent neonatal diabetes mellitus (PNDM), a much more serious condition than young-onset T2DM, is brought on by heterozygous inactivating gck mutations (MODY2) [35-37]. On the other hand, there is a direct correlation between the level of enzyme activation and the severity of persistent hyperinsulinemic hypoglycemia of infancy (PHHI), which is caused by gain-of-function activating gck mutations [35]. Therefore, we hypothesized that phytocompounds from Coleus amboinicus could activate GCK activity for the conversion of glucose to glucose 6-phosphate after binding with amino acids of GCK, resulting in a decrease in blood glucose by increasing cellular utilization and, potentially, a future application in the treatment of T2DM.

Molecular docking is a simulation method that considers the best location for a molecule to bind to a protein binding site. In this approach, the binding site is selected in the 3D coordinate space of the target, and the binding affinity is computed based on the final orientation of the molecule within the binding site. The importance and sensitivity of binding affinity values are determined by the biggest large negative value (greatest binding affinity or lowest binding energy), which represents the most beneficial conformation of the complex created when the implicated ligand successfully binds with the active pockets of the target.

Additionally, by examining ligand binding modes and direction in the target protein's receptor pocket, the molecular docking model has been used to confirm the anti-diabetic efficiency of Coleus amboinicus compounds (Table 1). The molecular docking investigation was carried out using AutoDock Vina in PyRx 0.8. A molecular docking and virtual screening program with multicore and multithreading capabilities are called AutoDock Vina. The ligand-receptor affinity was measured using the binding free energy (G binding) value. The whole inter-molecular energy, total internal energy, and torsional free energy were added up to determine the binding free energy, which was then subtracted from the energy of the unbound system. The optimal interaction site was determined using the conformation with the least energy binding value.

**Figure 1:**
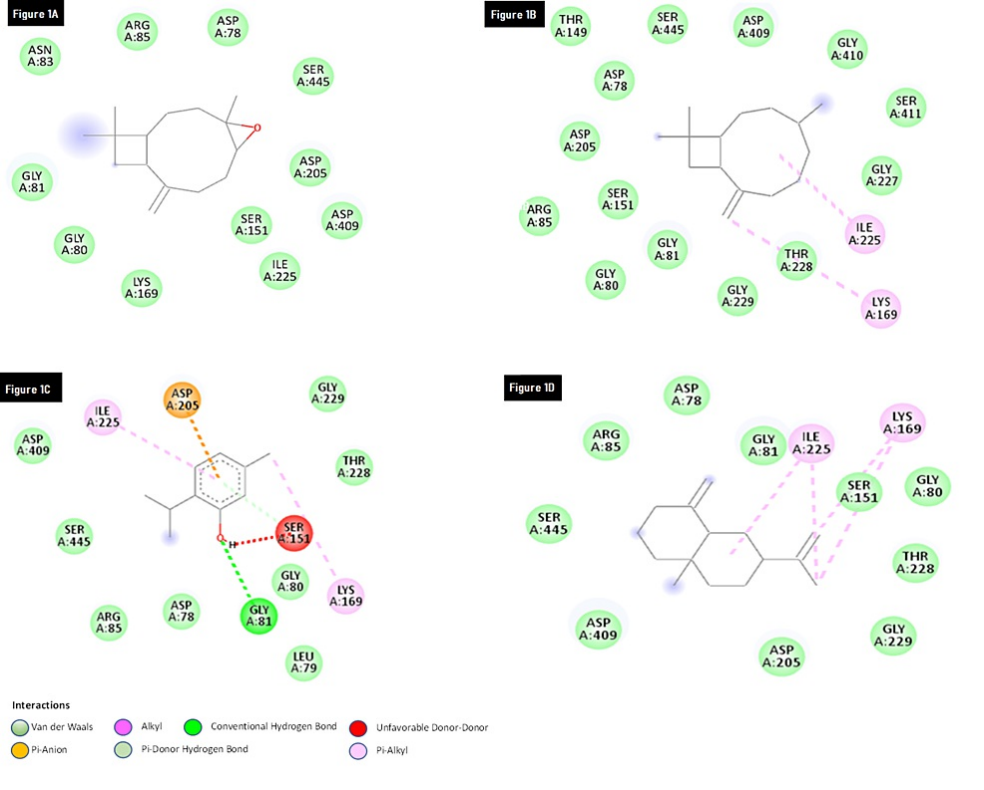
Molecular interaction of glucokinase with (Figure 1A) Caryophyllene_oxide; (Figure 1B) beta-Caryophyllene; (Figure 1C) Thymol; (Figure 1D) beta-Selinene

**Figure 2:**
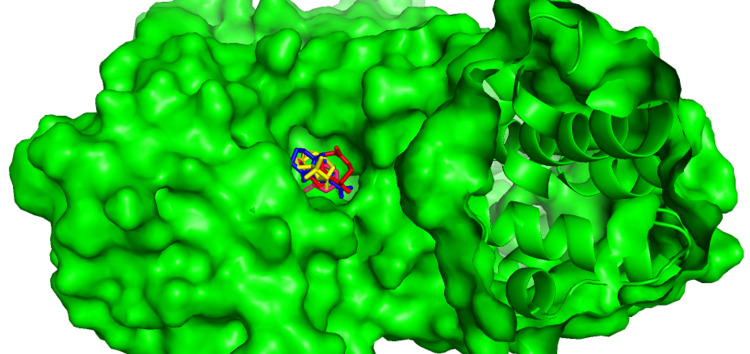
Molecular surface representation of glucokinase with selected compounds: beta-Caryophyllene (red), beta-Selinene (blue), Caryophyllene_oxide (Yellow), Thymol (Pink)

Figure 1A showed the interaction between glucokinase with Caryophyllene oxide. Compared to other ligands, it showed strong binding with glucokinase in terms of binding energy. This complex showed the binding energy of -6kal/mol, but it does not show any type of interaction with glucokinase protein. Figure 1B showed the interaction between the beta-Caryophyllene with glucokinase. It showed a binding energy of -5.7 kcal/mol and it formed the two pi-alkyl interactions with the amino acid residues of LYS-169 and ILE-225. Figure 1C showed the interaction of Thymol with glucokinase. It formed the four interactions with the binding site of glucokinase. The amino acid GLY-181 form the hydrogen interaction with thymol, and the amino acids like ASP-205, LYS-169, and ILE-225 form the pi-alkyl interactions with glucokinase. Figure 1D showed the interaction between the beta-Selinene and glucokinase. It showed good binding with a binding energy of -5.3kcal/mol. From these results, we observed that all the compounds formed the pi-alkyl interaction with the amino acids of LYS-169 and ILE-225. The several Pi-sigma interactions (Pi-alkyl and Pi-Sulphur), the majority of which involve charge transfer, aid in intercalating the medication in the receptor's binding region. There is an interaction between the pi-electron cloud above an aromatic group and the electron group of any alkyl group in pi-alkyl interactions Figure 2. The presence of these types of interactions in docking studies confirmed that selected compounds had a strong binding affinity with the target protein.

As determined by assessing their bonding contacts in terms of H-bond, hydrophobic interactions, and G of the best docked poses, majority of the drugs demonstrated considerable binding in the allosteric region of Glucokinase protein (Table 2). These compounds have a complementary fit in the allosteric region of the GCK protein, according to docking studies.

## Conclusions

Natural substances have long been used to cure and prevent illnesses in humans. Based on the aforementioned in silico investigation, we concentrated on the hydrogen and hydrophobic interactions in the position of ASP-205, LYS-169, GLY-181, and ILE-225 residues of the glucokinase protein, and the binding of naturally occurring molecules was seated appropriately on the particular location. As a result, the proposed compounds are offered to the scientific community for further research. The results of this study demonstrated that interactions between Coleus amboinicus compounds and the Glucokinase enzyme were revealed by in silico molecular docking investigations, demonstrating the need for additional research to develop potent glucokinase activators for the treatment of T2DM.
